# One Health and Cyanobacteria in Freshwater Systems: Animal Illnesses and Deaths Are Sentinel Events for Human Health Risks

**DOI:** 10.3390/toxins7041374

**Published:** 2015-04-20

**Authors:** Elizabeth D. Hilborn, Val R. Beasley

**Affiliations:** 1 National Health and Environmental Effects Research Laboratory, Office of Research and Development, United States Environmental Protection Agency, Research Triangle Park, NC 27711, USA; 2 Department of Veterinary and Biomedical Sciences, College of Agricultural Sciences, the Pennsylvania State University, University Park, PA 16802, USA; E-Mail: vbeasley@psu.edu

**Keywords:** harmful algae, cyanobacteria, blue-green algae, human, animal, sentinel event, ecosystem, health, one health, biotoxin, cyanotoxin

## Abstract

Harmful cyanobacterial blooms have adversely impacted human and animal health for thousands of years. Recently, the health impacts of harmful cyanobacteria blooms are becoming more frequently detected and reported. However, reports of human and animal illnesses or deaths associated with harmful cyanobacteria blooms tend to be investigated and reported separately. Consequently, professionals working in human or in animal health do not always communicate findings related to these events with one another. Using the One Health concept of integration and collaboration among health disciplines, we systematically review the existing literature to discover where harmful cyanobacteria-associated animal illnesses and deaths have served as sentinel events to warn of potential human health risks. We find that illnesses or deaths among livestock, dogs and fish are all potentially useful as sentinel events for the presence of harmful cyanobacteria that may impact human health. We also describe ways to enhance the value of reports of cyanobacteria-associated illnesses and deaths in animals to protect human health. Efficient monitoring of environmental and animal health in a One Health collaborative framework can provide vital warnings of cyanobacteria-associated human health risks.

## 1. Introduction

### 1.1. Freshwater Cyanobacteria

Freshwater cyanobacteria and their toxins (cyanotoxins) pose risks to human and animal health via contamination of water sources and aquatic communities globally. Dense accumulations of cyanobacterial cells, or colonies are termed ‘blooms’ and these occur most commonly, but not exclusively, in nutrient-rich, warm, bodies of water with little movement or mixing among layers [1]. When cyanotoxins are produced, or when cyanobacterial biomass and/or cyanotoxins disrupt ecological processes, the events are loosely termed ‘harmful algal blooms’ (HABs). The World Health Organization has developed guidelines based upon cyanobacterial cell densities in water, and advises that the presence of dense scums near bathing areas may indicate substantial human health risks [2]. Although cyanobacteria are naturally-occurring, anthropogenic activities now contribute to increased occurrence of HABs globally [3]. Nutrient pollution from human and animal wastes that wash into surface waters, fertilizer applications, atmospheric nutrient deposition, burning of plant material, overgrazing, warmer weather, drought conditions that reduce terrestrial plant uptake of nutrients as well as reduce the depth and flow of water bodies all contribute to bloom formation [4]. Conditions that promote cyanobacteria occurrences are expected to increase based upon model projections of future human population growth, land use patterns and climate change [5].

Many genera of cyanobacteria produce potent toxins as secondary metabolites, some of which are released before, and others largely after, cyanobacterial lysis. The evolutionary value of cyanotoxins to their producers is not fully characterized, although benefits may include: quorum sensing, grazer deterrence, a potential competitive advantage in aquatic environments via allelopathy toward other cyanobacteria, and improved regulation of intracellular phosphate or electrolyte concentrations [6,7,8]. Cyanobacteria often produce foul taste and odor compounds such as geosmin and 2-methylisoborneol during their life cycle, senescence and decomposition [9,10]. These taste and odor compounds are not believed to present major health risks, but their potential toxicity has been little studied [11]. Importantly, these odorous compounds can indicate the need to prevent human and animal exposure to water that may also contain potentially lethal cyanotoxins [9].

During the 19th and most of the 20th century, the toxicity of water samples was assessed primarily by the use of the animal bioassay. Francis, in 1878 authored one of the first reports of the toxicological assessment of a harmful cyanobacterial bloom and the intentional use of an animal as an indicator of human health risk [12]. During the last 100 years, advances in investigative and diagnostic tools have helped characterize cyanobacteria and cyanotoxins, including: light microscopy with increasingly refined phycology and cyanobacterial taxonomy; and the identification and characterization of some cyanotoxins using molecular, chemical and biochemical assays [13,14]. During the latter part of the 20th century, over 100 unique cyanotoxins have been identified, and new compounds continue to be isolated, and structurally characterized. Multiple functional classes of cyanotoxins have now been described, including: hepatotoxins, neurotoxins, dermatotoxins and cytotoxins [15,16,17,18]. Examples of hepatotoxins include the cyclic peptides: microcystins and nodularins, although their effects are broader than liver toxicity alone. Cylindrospermopsin is a potent sulfated tricyclic guanidine cytotoxin with bioactive metabolites. Cyanobacterial neurotoxins include saxitoxin and neosaxitoxin, which are complex alkaloid sodium channel blockers, the cyclic alkaloid nicotinic agonists anatoxin-a and homoanatoxin-a, and the organophosphorus cholinesterase inhibitor anatoxin-a(s). Lyngbyatoxins are cyanobacterial dermatotoxins that occur in fresh, brackish, and marine waters. Cyanobacteria also produce lipopolysaccharides, which are general irritants [18]. No cyanotoxins are fully characterized for toxicity, for geographic occurrence, or for the environmental conditions necessary and sufficient for their production. Concerted research efforts are underway in many countries to characterize the occurrence of harmful cyanobacteria and their effects on ecosystem functions as well as human and animal health. However, the number of cyanotoxins and combinations of cyanotoxin mixtures in the environment complicates risk assessments focused on potentially harmful cyanobacterial exposures. 

### 1.2. The Concept of “One Health”

“One Health” is a term that is increasingly being used in the early 21st century to convey how health rests upon interdependent collaborations among professionals in human and animal health, and wildlife and environmental sciences [19,20]. Nevertheless, the concept of One Health as a more inclusive and holistic way to study and maintain health within a cross-species continuum is ancient. Zinsstag *et al*. (2011) provided overviews of the origins, scope, systems thinking, and value of One Health, including how China’s Zhou Dynasty (11th–13th century) organized public health systems involving both medical doctors and veterinarians [21]. In the 1960s, Rachel Carson made connections between the application of highly toxic pesticides and adverse effects on human, domestic animal, and terrestrial and aquatic wildlife health. A One Health approach would have benefitted all of those involved in an incident of methylmercury intoxication in Japan in the 1950s [22]. A prolonged industrial release of methylmercury into waters off the coast of Minamata poisoned first fish and then birds; later cats and thousands of humans were sickened as the toxin spread throughout the food web. One Health insights relevant to infectious diseases were relied upon by such scientists as Edward Jenner, when, in developing a vaccine against smallpox (a human disease), he demonstrated the cross reactivity of human antibodies between smallpox and the less pathogenic cowpox (an animal disease). Countless other scientists, clinicians and public health practitioners have operated in a One Health paradigm in the pursuit of control of zoonotic disease, in food and water safety, as well as in environmental protection to optimize the health and wellbeing of earth’s biota. In short, One Health is a paradigm that recognizes the interdependence of human, animal, plant, microbial, and ecosystem health [23]. Effective multidisciplinary research, surveillance, and stewardship are essential for synchronous improvements in the health of humans, other animals, plants and ecosystems [19]. Recently, funding for public health from public and private sources has declined [24,25]. Unfortunately, this has coincided with reductions in essential public health services provided by functional ecosystems [26]. The One Health framework offers an interdisciplinary paradigm that seeks to optimize health by leveraging existing resources and capabilities among human, veterinary and ecosystem health experts to address some of the most the complex, multidisciplinary challenges that define the 21st century.

Reports of human health and animal health tend to be published separately and are discussed separately. However, harmful cyanobacteria impact both humans and animals. Animals often experience direct, high-intensity exposures to harmful cyanobacteria which result in illnesses and deaths. Cyanobacteria-associated animal illnesses or deaths can therefore be used to warn of risks and if heeded, action may be implemented to avoid adverse human health effects [27]. Our goal is to provide a representative overview of the adverse effects of harmful freshwater cyanobacteria on humans and other vertebrates and to comprehensively review reports that include incidents where animal illnesses and deaths have served as sentinel events to warn of potential or actual human health risks.

## 2. Selected Human Health Reports

Human health may be adversely impacted by harmful cyanobacteria from many sources, and via multiple routes of exposure. The highest impact outbreaks of cyanobacteria-associated intoxications and deaths have been reported when patients requiring hemodialysis were directly exposed to cyanotoxins intravenously via dialysate prepared from contaminated water [28,29]. This route of exposure to cyanotoxins resulted in toxic hepatitis, multi-organ damage and death [28,29,30,31].

People are most frequently exposed to harmful cyanobacteria via contaminated water. People may be exposed orally, dermally and occasionally by aspiration to aquatic microbial communities containing cyanobacterial cells and mixtures of cyanotoxins during recreational activities on or in untreated surface waters [32,33,34,35,36]. Occasionally, these exposures have resulted in severe respiratory impairment characterized by pneumonia and adult respiratory distress syndrome [32,35]. Less severe effects include fever, other respiratory illness, signs and symptoms of respiratory and dermal allergy, and dermatologic, gastrointestinal, neurologic, otic, and ocular signs and symptoms [33,34,36,37,38,39,40,41,42,43]. Occupational exposures to harmful cyanobacteria have been reported after routine work on an incidentally contaminated surface water body, in relation to investigation of a cyanobacterial bloom, and following an investigation of cyanobacteria-associated animal illnesses and deaths [34]. 

Drinking water contaminated with harmful cyanobacteria has been associated with liver and kidney damage [44,45], and rarely, severe illness, extended hospitalizations and deaths have occurred [45,46,47]. Acute health effects such as gastroenteritis, muscle pain and dermatitis associated with home use of contaminated drinking water have been reported [43,48]. When municipal systems have been contaminated, large numbers of people may be exposed and become ill [49,50,51,52,53]. The International Agency for Research on Cancer has determined that, while current data on microcystins and nodularins are inconclusive in regard to human carcinogenesis, promotion of liver tumors by these toxins is plausible [54]. Zhou [55] reported that use of potentially microcystin-contaminated drinking water supplies was associated with higher rates of colorectal cancer in human populations in parts of China. Conventional drinking water treatment involving filtration, flocculation, and disinfection reduces, but does not always eliminate cyanobacteria and cyanotoxins. More sophisticated methods may be required to reduce cyanotoxins in finished drinking water to acceptable concentrations [56]. However, drinking water treatment processes may become impaired or ineffective when large quantities of cyanobacterial biomass enter the source water intake.

Some poorly characterized human health risks include: repeated voluntary exposure to cyanotoxins through ingestion of cyanobacteria (blue green algae) as food or as supplements [57,58]; involuntary exposure via inhalation of cyanotoxins during activities on or in contaminated waters, and ingestion of contaminated aquatic animal foods or vegetables grown with contaminated irrigation water [59,60,61,62].

## 3. Selected Animal Health Reports

HABs and associated adverse animal health impacts have been recorded for over 180 years. The first published report believed to document a harmful cyanobacteria bloom was by Hald to the Danish government in 1833. Hald described cattle and fish deaths associated with ‘sick’ lakes where green material covered the surface of the water. The bloom material itself was uncharacterized. Hald wrote that he did not know if the green substance was “…caused by water plants, insects or minerals…” [63].

Most early reports of animal deaths associated with harmful blooms were circumstantial. Waters were initially suspected of being harmful because of the temporal and spatial proximity of dead and dying animals observed in and around a bloom. Although the toxicity of these aquatic ‘plant’ materials was surmised by observation of associated animal deaths, the toxigenic organisms and the toxic principles themselves were uncharacterized. Francis was the first to scientifically investigate the toxic effects of a cyanobacterial bloom [12]. After mass livestock deaths in Lake Alexandrina in Australia, he administered a sample of the *Nodularia spumigena* bloom material from the lake to a sheep. He then compared necropsy results of the animal experimentally exposed with sheep that had died following natural exposure to the bloom and concluded that cyanobacteria were the source of the toxic effects.

Harmful cyanobacteria adversely affect wildlife, livestock and companion animals. Schwimmer and Schwimmer [64] compiled and summarized over 65 wildlife, livestock and domestic animal mortality events associated with cyanobacteria during 1878–1960. Animal deaths associated with harmful cyanobacteria have been reported from Europe, North America, South America, Australia, Africa and Asia [12,65,66,67,68,69]. Stewart, *et al.* [70] provided a selective review of published reports of cyanobacteria-associated animal morbidity and mortality events from around the world, with representative case studies of livestock, companion animal and wildlife deaths.

Reports of livestock deaths following exposures to cyanobacteria under field conditions have been reported from every inhabited continent and involved ruminants, hogs, horses, fowl, cultured fish and even honeybees [65,66,71,72,73,74,75]. Livestock with access to farm ponds and portions of lakes may be at risk of exposure during a bloom, especially when wind-driven surface blooms accumulate at the site of animals’ water access. Overflow of bloom material from farm ponds that contaminates animals’ pasture can also be a source of poisoning for livestock [76]. Antemortem signs of intoxication vary and are dependent upon the cyanotoxins, the dose and time frames involved, the therapeutic interventions employed and individual characteristics of the exposed animals. Acute effects often include: hypersalivation, agitation, anorexia, pale mucus membranes, weakness, dyspnea, recumbancy, depression, ataxia, diarrhea, muscle tremors and fasciculations, convulsions, apparent blindness and sudden death [77,78,79]. Birds may display weakness and neurologic signs such as ataxia, and hyperextended necks (opisthotonos) prior to death. Cyanobacteria have been associated with mass mortality events in catfish and carp cultured in ponds; microcystins in water were accompanied by clinical signs of illness and gross lesions in the liver [73,75]. Because of microcystin residues, the latter authors (Singh and Asthana) cautioned against human consumption of the tissues of contaminated fish.

Reports of cyanobacteria-associated companion animal illnesses and deaths have most often involved dogs. Dogs have been observed consuming scums of cyanobacteria that accumulate near the shore, drinking contaminated water, and licking bloom material from their hair coats after wading or swimming [80]. A recent summary of cyanobacteria-associated dog deaths in the United States compiles over 100 reports over the last 80 years [81]. The frequency of reporting of these events has greatly increased since the 1970s, however, reporting, attribution and detection biases were all factors that influenced the number of events that were confirmed as being associated with cyanobacteria during the study period. Deaths among other companion animal deaths such as cats are rarely reported [77]. Acute effects among companion animals include: vomiting, diarrhea, profuse salivation, weakness, convulsions, hemorrhage and sudden death [82,83].

Wildlife deaths associated with harmful freshwater cyanobacterial blooms are commonly reported, but undoubtedly many occur and are unreported because of the lack of human observation of the event. Multiple types of vertebrates may be harmed, from fish to birds to mammals [84,85,86]. In some instances it is not always possible to attribute wildlife deaths to harmful cyanobacteria because when the affected animals are found, they are too decomposed for reliable pathological and toxicological analyses. Fish and water birds are at especially high risk of harmful cyanobacteria-associated effects, and mass mortality events have been reported from most continents [66,68,85,87,88]. Cyanobacteria blooms may have direct and indirect adverse effects on fish and water birds. Direct intoxication may occur after exposure to harmful cyanobacteria, or cyanotoxin-contaminated food and water. Indirect effects of cyanobacterial blooms include a decrease in dissolved oxygen and the proliferation of *Clostridium botulinum* [89]. Large mortality events have occurred when birds are poisoned by botulinum toxin in aquatic environments [90,91].

## 4. Results: Animal Illnesses and Deaths that Served as Sentinel Events for Cyanobacteria-Associated Human Health Risks

Our search of the scientific literature yielded 18 reports describing at least 29 events where there were actual or potential cyanobacteria-associated human health risks accompanied by observations of sick or dead animals that were exposed to harmful cyanobacteria (Table 1). These episodes of human health risks have occurred among drinking water consumers, among workers investigating mass mortality events, and among recreational users of water bodies that were contaminated with cyanobacteria. Eleven of 18 reports describe animal illnesses or deaths that alerted authorities to the presence of contaminated water and warnings were issued and/or action was taken to prevent human exposure. 

**Table 1:** Reports of animal illnesses and deaths that served as sentinel events for cyanobacteria-associated human health risks.

Location; reference	Year	Number events	Cyanobacteria	Toxin	Animal illness	Human illness, exposure route	Interagency coordination
Lake Alexandrina, Australia; [92]	1878	>1	*Nodularia spumigena* identified in water	Unknown	Several hundred livestock deaths	Undescribed illness in one individual after drinking contaminated water	Investigation, warnings issued prior to human illness
Elk River, Kanawha River, Ohio River, West Virginia; Ohio River Ohio; Ohio River, Kentucky, United States; [50]	1930–1931	>6	*Anabaena flos-aquae* identified in water	Unknown	Fish deaths Kanawha River	Gastrointestinal illness among thousands of people receiving drinking water from rivers	Investigation, no known warnings issued
Storm Lake, Iowa, United States; [85]	1948	>1	*Anabaena flos-aquae* identified in water	Unknown	Fish, dogs died	None reported	Investigation, warnings issued
Lake Dauphin, Manitoba, Canada; [77]	1951	1	*Aphanizomenon flos-aquae* identified in water	Unknown	Horse, dogs died	None reported	Investigation, warnings issued
Echo Lake, Qu’Appelle Lake, other lakes in Saskatchewan, Canada; [93,94]	1959	>2	*Anabaena circinalis* (identified in stool sample)	Unknown	Multiple livestock, fish, geese, dogs died	Gastrointestinal illness among individuals with recreational exposure to lakes	Investigation, warnings issued prior to human illness
Hegman Reservoir, Montana, United States; [95]	1977	1	*Anabaena* spp. and *Aphanizomenon flos-aquae* identified in water	Unknown	Cattle, dogs died	None reported	Investigation, warnings issued
Lakes in Pennsylvania, United States; [40]	1979	2	*Anabaena* spp. identified in water	Unknown	Dog illness	None reported	Investigation, warnings issued before dog illness
Lake in Montana, United States; [96]	1984	1	Unspecified bloom identified in water	Unknown	Cattle deaths	None reported	Investigation, warnings issued
Lake in Alberta, Canada; [97]	1985	1	Unspecified bloom identified in water	Unknown	Bats, ducks died	None reported	Investigation, warnings issued
Guandiana River in Portugal; [98]	1987	1	*Aphanizomenon flos-aquae* bloom identified in water	Unknown	Fish deaths	Gastroenteritis, dermatitis among those who consumed drinking water	None known
Lake—Rutland Water in Leicestershire, United Kingdom; [99]	1989	1	*Microcystis aeruginosa* bloom identified in water	Microcystin-LR	Dog and sheep deaths	Gastroenteritis, dermatitis among those who recreated in water	Investigation, no known warnings issued
Zeekoevlei Lake, and others, near or in Western Cape Province, South Africa; [100,101]	1994	4	*Nodularia spumigena* and *Microcystis aeruginosa* bloom identified in water	Nodularin, Microcystin-LR	Dog and livestock deaths	None reported	Investigation, warnings issued
Pond in Mymensingh, Bangladesh; [74]	2002	1	*Anabaena flos-aquae* and *Microcystis aeruginosa* bloom identified in water	Unknown	Fish and goat deaths	Rash, eye and ear irritation	Investigation, no known warnings issued
River Meuse, Venlo Municipality, Netherlands; [102]	2003	1	Unspecified cyanobacteria	Unknown	Fish and bird deaths	Rash	Investigation, no known warnings issued
Buccaneer Bay Lake, multiple other lakes, Eastern Nebraska, United States; [42]	2004	>3	*Anabaena*, *Microcystis*, *Oscillatoria*, *Aphanizo-menon*	Microcystin-LR and microcystins	Dog, livestock, wildlife deaths	More than 50 reports of rash, skin lesions, headache and/or gastroenteritis	Investigation, warnings issued
Lakes, Ohio, United States; [36]	2010	2	*Anabaena* spp., *Cylindro-spermopsis raciborskii*, *Aphanizomenon* spp., *Planktolyngbya limnetica*	Microcystins, Anatoxin-a cylindrospermop-sin, saxitoxins	Dog, fish deaths, bird illness	Multiple effects including dermatologic, respiratory, neurologic illness and/or gastroenteritis	Investigation, no known warnings issued

In multiple instances, reports of animal deaths prompted investigations that led to the initial isolation and structural characterization of cyanotoxins from a water body without a specified use for drinking or recreation. One such report is Devlin *et al.* [103], who isolated and cultured a cyanobacterium associated with cattle deaths and determined the structure of anatoxin-a for the first time. In some instances, the toxic mechanism of action was first deduced in studies conducted after animals died in the field, and those mechanisms later were used for toxin isolation and structural elucidation. For example, after dog and other animal deaths were observed in a lake in South Dakota, United States, Mahmood *et al.* [104] detected a cholinesterase-inhibiting cyanotoxin for the first time. Subsequent studies based upon that biochemical mechanism led to characterization of anatoxin-a(s), the only known naturally occurring organophosphorous cholinesterase-inhibiting toxin [105]. 

Animal deaths have also been the trigger for the first regional detections of harmful cyanobacteria and specific cyanotoxins. Skulberg [87] raised awareness of problems from harmful cyanobacteria in Europe’s eutrophic waters by summarizing the findings of a 1980 survey performed in 26 European countries. At that time, eleven countries reported outbreaks of blue-green algae-associated animal intoxications. James [106] reported detecting anatoxin-a in Irish lakes for the first time during the summers of 1994 and 1995; the investigations followed reports of potentially toxic cyanobacteria-associated dog deaths at lakes during 1992 and 1993. Multiple reports of dog deaths in Scotland, New Zealand and France led to the finding of anatoxin-a in the absence of surface blooms; instead, the cyanotoxin was found to be associated with benthic cyanobacteria [83,107,108]. Mez *et al.* [109] reported isolation of microcystins from Swiss oligotrophic water for the first time after observing a pattern of cattle deaths.

## 5. Discussion

We found 18 reports of 29 or more events where animal illnesses and deaths served as potential sentinel events for human health risks related to harmful cyanobacteria [36,40,42,50,74,77,85,92,93,94,95,96,97,98,99,100,101,102]. Among 15 of 18 reports describing 25 or more events, cyanobacteria-associated animal illness clearly preceded any reports of human illness [36,42,50,77,85,92,93,94,95,96,97,99,100,101,102]. Among 11 of 18 reports describing 15 or more events, cyanobacteria-associated animal illness or death was recognized, authorities were alerted to the presence of contaminated water, and action was taken to warn people of the risk associated with exposure to potentially toxic water [42,77,85,92,93,94,95,96,97,100,101]. We found that among the instances where sentinel events were used to warn of the potential for human health risks there was effective communication among environmental, wildlife and health officials. 

We selectively described multiple additional reports of animal deaths that alerted investigators to the potential presence of harmful cyanobacteria, some even in the absence of an apparent surface bloom. These bodies of water were not reported as being used for human recreation or drinking water, so the risk for adverse human health effects was unclear [83,103,104,106,107,108,109]. However, the animal illnesses and deaths acted as sentinel events that warned of potential cyanobacteria-associated water toxicity.

Dogs, livestock and fish were most frequently cited as animals involved in sentinel events that warned of harmful cyanobacteria. Dogs in particular may be exposed to surface water bodies as they accompany people during recreation and some outdoor types of work. Dogs are likely to drink water they encounter and may ingest algal scums that are present. Dogs acted as sentinels of human health risk in 17 or more events, these events involved primarily recreational water sources, and one event described a poisoning at a potential drinking water source [36,40,42,77,85,93,94,95,99,100]. Livestock historically have been given access to ponds or lakes that people may use for recreation or drinking water. Types of livestock reportedly involved in sentinel events included primarily cattle and horses; fewer reports involved pigs and smaller ruminants. Livestock acted as sentinels of human health risks in 15 or more events, nine of these were described at recreational water sources, and at least two were described at potential drinking water sources [42,74,77,92,93,94,95,96,99,101]. Fish are raised in ponds as livestock, but also are free-living in lakes, rivers, and other water bodies. Fish acted as sentinels of human health risks in at least 14 events, seven of these were described at recreational waters, and seven were described at drinking water sources [36,50,74,85,93,98,102].

**Figure 1:**
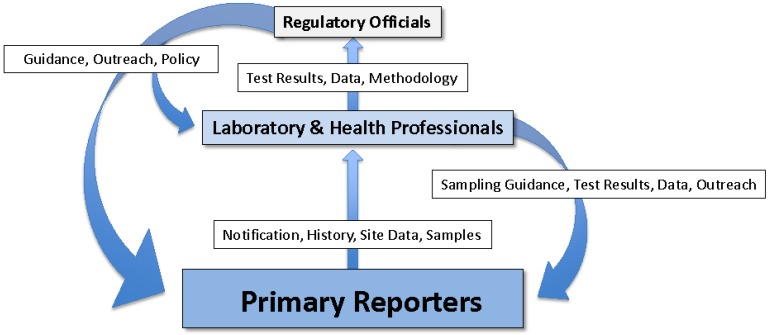
Functional groups and flow of information in a model One Health approach to harmful cyanobacteria identification, risk characterization and response. **Primary Reporters** include: Those who live, recreate or work on or near cyanobacteria-impacted water bodies such as: residents of water front homes; animal owners; lake and waterkeepers; environmental professionals; wildlife professionals; water management and utility personnel; fishermen; public safety personnel. **Laboratory and Health Professionals** include: Chemists, phycologists, wildlife biologists, agricultural specialists, toxicologists, veterinary pathologists, veterinarians and human health care providers. **Regulatory Officials** include: Public health, environmental health, environmental management, wildlife and agricultural personnel.

History has shown that the presence of dead or moribund animals in and around a body of water has served as a warning of potential human health risk associated with the water [63]. However, animal illnesses and deaths only effectively warn of human health risks if they are actually seen and the risk recognized. Therefore, it is important for animal deaths potentially associated with harmful cyanobacteria to be investigated and warnings to be issued to potential water users in a timely manner. Ideally, information should be transmitted from those who first encounter dead or dying animals, to wildlife or environmental stewards, veterinarians and public safety personnel. Samples of water and affected animals should be evaluated by laboratory and veterinary professionals. People with health effects should be referred to health care providers. The results of clinical and laboratory analyses should be communicated to investigators and primary reporters as well as to regulatory officials. Regulatory officials should develop monitoring programs and incident response protocols in concert with water and wildlife agencies, and develop and distribute educational materials and perform outreach to those potentially impacted by harmful cyanobacteria with the goal of risk reduction. Clinical care providers should be provided with information on sources and health impacts of harmful cyanobacteria as well as any established diagnostic methods and criteria. Therefore, wildlife, veterinary, medical, water management, laboratory and public health officials may all be potentially involved in a successful response to the occurrence of harmful cyanobacteria (Figure 1) [77,85,93,94]. 

Unfortunately, communication among these groups is not always frequent or timely, suggesting that there may be significant professional, structural and institutional barriers to adopting the widespread use of sentinel events to protect human health [110]. Auf der Heide notes that in smaller communities, where few public professionals are employed, professionals from multiple disciplines may actually interact more frequently and perform more efficiently and cohesively in the face of unusual or emergent health events [110]. In larger communities with frequent cyanobacterial blooms, providing the opportunity for intentional communication and collaboration activities among these groups before concerted action is needed may be rewarded by more efficient human, animal and environmental health protection.

The One Health initiative provides a framework for multidisciplinary interaction and cooperation among specialists in human, animal and environmental health [19]. This approach has most frequently been applied to the identification and control of zoonotic diseases, but there is a need for coordination among professionals who normally focus solely on human, domestic animal, or wildlife/ecological toxicology as well [111,112]. Commonly-occurring environmental contaminants such as harmful cyanobacteria are human health hazards for which a One Health approach is being successfully applied. A number of the reports of integrated surveillance and preventive measures mentioned above attest to the value of attention to multiple species groups, but these are largely “after-the-fact” responses, when what is needed is to be more proactive. 

Nations in Europe have developed One Health guidelines to reduce health risks associated with harmful cyanobacteria. For example, in Scotland, a set of guidelines for risk assessment of cyanobacteria-impacted waters has been developed and updated with the goal of protecting both human and animal health [113]. The responsibility to initially assess the potential risks to human and animal health from harmful cyanobacteria has been assigned to water quality officials and workers, public and environmental health officials and interested individuals. Guidelines for incident investigation, reporting and deployment of warnings to the public have been developed. France has developed the SAGIR (*surveiller les maladies de la faune sauvage pour agir*), a surveillance network for zoonotic disease and environmental toxins which includes the *Fédérations des chasseurs* and *l’Office national de la chasse et de la faune sauvage*. Hunters report wildlife mortality events to SAGIR, which then prompts wildlife personnel to investigate the animal death(s) and accompanying environmental conditions. This helps to maximize the early detection of emerging health threats, informs risk assessment and ultimately contributes to the protection of animal and human health [114]. 

In the United States, the Centers for Disease Control and Prevention (CDC), National Center for Environmental Health had historically provided funding to states to collect reports of cyanobacteria-associated human and animal health, but funding has ended and not all member states are able to continue related activities [81]. Currently, efforts are underway to incorporate reports of animal and human illness associated with harmful algal blooms into the CDC National Outbreak Reporting System (NORS), a voluntary national system that receives reports of food-borne, waterborne and other outbreaks of human illness [115]. Although the incorporation of human and animal-related illnesses and deaths into NORS will raise general awareness about harmful cyanobacteria among some state health officials, it is currently too early to see if these efforts will be successful in fostering integration and communication among human, animal and environmental health specialists that will lead to a One Health collaborative framework for integrated health protection.

### Limitations and Sources of Uncertainty

Cyanobacteria blooms do not always produce toxins, and cyanobacteria and cyanotoxin concentrations are heterogeneous spatially and temporally [116]. Acute animal illnesses and deaths are useful as indicators of human health risks, but harmful cyanobacterial blooms may exist in the absence of dead or impaired animals [117]. Therefore, the absence of animal illness and death at a water body should not be interpreted that no risk, or minimal human health risk exists. 

Published reports of cyanobacteria-associated animal death and illness are relatively uncommon. Many events are inaccessible to the public as they remain in the notes and files of wildlife officials, public and environmental health personnel, and veterinarians. Many cases of suspected cyanotoxin poisoning are not confirmed because of the limited availability and cost of cyanotoxin analyses. An expanded review and report of all data sources including those: presented through the broadcast media, published in print, on-line and in newsletters, and stored in medical (human health and veterinary) records, and records of public, environmental health and wildlife officials would likely augment the total number of recognized events where animals have acted as sentinels for cyanobacteria-associated human health risks. 

For an animal death to serve as a sentinel event of cyanobacteria-associated risk, people must observe the animal’s remains in a timely manner. Carcasses of smaller species will not necessarily persist in the environment. Such deaths, especially if few animals are involved, may often be missed by human observers. Conversely, large numbers of smaller animals that are obvious to the observer, large animals whose carcasses persist in the environment, and human-affiliated animals such as companion animals or livestock are more likely to be observed and recorded. 

When dead animals are observed, prompt notification of veterinary, wildlife or health officials is needed to determine a cause for the deaths. However, a prompt post-mortem examination alone is insufficient to conclude that a death is associated with harmful cyanobacteria [118]. Chen *et al.* suggested that analysis of the contents of the gastrointestinal tract may provide a useful indication of the recent composition of cyanobacteria at the collection site [119]. Animals appear to bioaccumulate cyanotoxins at different rates so that tissue concentrations evaluated at the time of death may not reflect current environmental conditions at the collection site [120]. Animals of different species also vary in susceptibility to certain cyanotoxins, but the basis of this variation remains to be well characterized [121].

Cyanobacteria may persist in water bodies and may recur at a given site. However, a previous mass mortality event correctly attributed to cyanobacteria and cyanotoxins, does not mean that a subsequent event at the same site has the same causation. For example, mass bird and fish mortality events in Donaña National Park, Spain in 2001 were confirmed to be associated with microcystins [122], but mass bird deaths at the same site in 2003 were eventually attributed to an infectious etiology, *Pasteurella* spp. [123].

Some freshwater biotoxins have not been included in this review due to insufficient information about human health risks. These include: emerging toxins such as β-methylamino-ʟ-alanine (BMAA), or toxins that are currently only associated with animal toxicity such as the recently reported causative agent of avian vacuolar myelinopathy (AVM) [124,125]. However, these contaminants may potentially play a larger role in future reports of outbreaks of illness that involve both animals and humans. 

## 6. Conclusions

This report illustrates how the recognition of and response to cyanobacteria-associated animal illnesses and deaths may be used to reduce the risks associated with human exposures to harmful cyanobacteria. Using a One Health approach is an efficient way to manage an environmental risk that involves multiple disciplines and professional specialties. Currently, barriers to maximizing the value of animals as sentinels in this context include: under reporting; limited resources for surveillance and investigation of events; and the potential lack of routine means of communication among potential One Health partners in environmental health, environmental management, human and veterinary medicine [111]. To enhance the value of animal sentinel events for harmful cyanobacteria-associated human health risk, we recommend:

- Engaging public health, domestic animal health, wildlife and ecosystem health personnel in group training and communication exercises.
- Developing improved methods to support identification and quantification of harmful cyanobacteria in water sources and analyses of cyanotoxins in cyanobacteria, water, and biological samples from exposed animals and humans.
- Including reports of harmful algal blooms and associated human and animal illness in health and environmental surveillance systems.
- Using successful models such as the Scottish risk assessment reporting guidelines and SAGIR to improve harmful cyanobacteria recognition and response in other nations around the world [113,114].

Such actions, combined with greater global awareness of harmful algal blooms as a multidisciplinary problem should increase the utility of reports of animal illnesses and deaths to inform the characterization of human health risks.

## 7. Materials and Methods

We searched PubMed and Web of Science databases for the terms: “(cyanobacter* AND animal)”; “(blue-green algae AND animal)”; “(cyanobacter* AND human)”; “(blue-green algae AND human)”; “(cyanobacter* AND health)”; “(blue-green algae AND health)”. We restricted these records to: (1) reports of animal exposures to cyanobacteria, cyanotoxins or uncharacterized freshwater blooms and evaluated these to determine if there was a human health or ambient human exposure component also described; (2) among reports of cyanobacteria-associated human illness, we assessed if there was animal death or illness also reported from the site of exposure. When more than one report described a single event, we chose the most comprehensive report(s) of human health exposure and effects. References of all reports were examined to identify other applicable reports to include in the review.
